# Integrative Multi-Omics Analysis of Identified NUF2 as a Candidate Oncogene Correlates With Poor Prognosis and Immune Infiltration in Non-Small Cell Lung Cancer

**DOI:** 10.3389/fonc.2021.656509

**Published:** 2021-06-10

**Authors:** Mengqing Chen, Shangkun Li, Yuling Liang, Yue Zhang, Dan Luo, Wenjun Wang

**Affiliations:** ^1^ Department of Respiratory and Critical Care Medicine, The Affiliated Hospital of Southwest Medical University, Luzhou, China; ^2^ Department of Anesthesiology, The First Affiliated Hospital of Chongqing Medical University, Chongqing, China; ^3^ Department of Respiratory and Critical Care Medicine, Southwest Medical University, Luzhou, China

**Keywords:** NUF2, non-small cell lung cancer, prognosis, immune infiltration, multi-omics

## Abstract

**Background:**

Lung cancer is one of the most common malignant tumors and the leading causes of cancer-related deaths worldwide. As a component of the nuclear division cycle 80 complex, NUF2 is a part of the conserved protein complex related to the centromere. Although the high expression of NUF2 has been reported in many different types of human cancers, the multi-omics analysis in non-small cell lung cancer (NSCLC) of NUF2 remains to be elucidated.

**Methods:**

In this analysis, NUF2 expression difference analysis in non-small cell lung cancer was evaluated by Oncomine, TIMER, GEO, and TCGA database. And the prognosis analysis of NUF2 based on Kaplan-Meier was performed. R language was used to analyze the differential expression genes, functional annotation and protein-protein interaction (PPI). GSEA analysis of differential expression genes was also carried out. Mechanism analysis about exploring the characteristic of NUF2, multi-omics, and correlation analysis was carried out using UALCAN, cBioportal, GEPIA, TIMER, and TISIDB, respectively.

**Results:**

The expression of NUF2 in NSCLC, both lung adenocarcinoma (LUAD) and squamous lung cancer (LUSC), was significantly higher than that in normal tissues. The analysis of UALCAN database samples proved that NUF2 expression was connected with stage and smoking habits. Meanwhile, the overall survival curve also validated that high expression of NUF2 has a poorer prognosis in NSCLC. GO, KEGG, GSEA, subcellular location from COMPARTMENTS indicated that NUF2 may regulate the cell cycle. Correlation analysis also showed that NUF2 was mainly positively associated with cell cycle and tumor-related genes. NUF2 altered group had a poorer prognosis than unaltered group in NSCLC. Immune infiltration analysis showed that the NUF2 expression mainly have negatively correlation with immune cells and immune subtypes in LUAD and LUSC. Furthermore, quantitative PCR was used to validate the expression difference of NUF2 in LUAD and LUSC.

**Conclusion:**

Our findings elucidated that NUF2 may play an important role in cell cycle, and significantly associated with tumor-related gene in NSCLC; we consider that NUF2 may be a prognostic biomarkers in NSCLC.

## Introduction

Lung cancer has a high incidence in the worldwide. The 5-year survival rate of lung cancer patients mainly depends on the stage of the disease and regional differences, with fluctuations ranging from 4% to 17% (1). Non-small cell lung cancer (NSCLC) and small cell lung cancer (SCLC) are the most commonly used diagnostic terms for lung cancer (2). NSCLC are approximately 80% to 85% of lung cancers, which contains approximately 40% to 50% cases of lung adenocarcinoma (LUAD) and 20% to 30% cases of lung squamous cell carcinoma (LUSC) (3). Chemotherapy is the most commonly therapy used to treat lung cancer, but its effectiveness is limited, while immunotherapy is currently expensive, therefore, targeted therapy is currently the better choice for most patients. Up to present, the research progress of NSCLC has found many target genes that correlated with tumor metastasis, invasion, prognosis, such as EGFR, KRAS, ALK, ROS1, etc. However, since patients with EGFR, ALK, ROS1 gene mutations currently account for only a portion of patients with non-small cell lung cancer, and some mutations have few drug options, many patients have developed drug resistance after targeted drug therapy, there is an urgent need to discover new potential biomarker for NSCLC (4).

In the process of mitosis, chromosomes must be accurately allocated to progeny cells. If the DNA copy in a cell is incomplete or DNA is damaged, it will lead to genetic disorders and diseases, such as cancer (5). The nuclear division cycle 80 (NDC80) complex is a heterotetrameric protein complex, and NUF2, as an important part of the NDC80 complex, is essential for kinetochore-microtubule attachment and chromosome separation. Previous studies have shown that NUF2 may contribute to the kinetochore-microtubule attachment in mammalian meiosis, it is a critical regulator of meiotic cell cycle progression in mammalian oocytes (6). Previous evidence demonstrate that NUF2 plays as a prognostic biomarker and therapeutic target in hepatocellular carcinoma, breast cancer, and oral cancer (7–9), and NDC80 complex gene might be an early indicator of diagnosis and prognosis of lung adenocarcinoma (10). These studies have shown that NUF2 might be a candidate biomarker and therapeutic target in cancer with great potential, but the role of the NUF2 gene in NSCLC has not been systematically explored in multiple aspects.

In recent years, more and more platforms, databases, and various data sets have enabled cancer researchers to use multi-omics data conducting bioinformatics analysis of cancer. In order to get a better understanding of the roles of the NUF2 gene in NSCLC, we perform expression profiling, prognosis valuation, DNA methylation, gene mutation, immune infiltration, clinic pathologic association significance of the NUF2 gene with the public-accessible cancer genomics database and also validated NUF2 expression by reverse transcription polymerase chain reaction (RT-PCR) in NSCLC.

## Materials and Methods

### Cell Culture

Human bronchial epithelial cells (16HBE), human lung adenocarcinoma cell line (A549), and human lung squamous cell line (H520) were obtained from American type culture collection (ATCC, USA). 16HBE cell line was maintained using Kerotinocyte Medium (Sciencell, USA). A549 cell line was maintained in Dulbecco’s Modified Eagle Medium (Biological Industries, Israel) with 10% fetal bovine serum (Gibco; Thermo Fisher Scientific, USA). H520 cell line was maintained in RPMI-1640 Medium (Biological Industries, Israel) which supplemented with 10% fetal bovine serum (Gibco; Thermo Fisher Scientific, USA). These three types of cell lines were cultured at 37°C with an atmosphere of 5% CO2. The mRNA expression levels of NUF2 were detected by RT-PCR.

### Gene Expression Analysis

We initially evaluated NUF2 transcription levels between NSCLC and normal tissues in pan-cancer from Oncomine (https://www.oncomine.org/) (11) and TIMER (https://cistrome.shinyapps.io/timer/), and then, we studied the transcription levels of NUF2 in LUAD and LUSC (*P* < 0.01). We selected GSE32863(LUAD) from the Gene Expression Omnibus (GEO, http://www.ncbi.nlm.nih.gov/geo/) and acquired data of LUSC from The Cancer Genome Atlas (TCGA, https://portal.gdc.cancer.gov/). Then, we selected GSE77803 to find the differential expression genes (DEGs) between NUF2 low-expression and high-expression patients. TOP20 differentially upregulated and downregulated expressed genes was next to assess correlation analysis.

### Prognosis Analysis and the Association of NUF2 Expression With Clinical Features in NSCLC

We assessed the effect of NUF2 expression on the survival rate in NSCLC and the subtype of it (LUAD and LUSC) through Kaplan Meier plotter (http://kmplot.com/). The association of NUF2 expression with clinical features was explored using the UALCAN database (http://ualcan.path.uab.edu/index.html) (12–14).

### Functional Enrichment Analysis and Protein-Protein Interaction

We next determined the functional annotation and protein-protein interaction network (PPI network) of the differential expressed genes above (including NUF2) in NSCLC. Gene ontology (GO) and Kyoto Encyclopedia of Genes and Genomes (KEGG) pathway enrichment analysis were utilized to evaluate the possible functions of the differential expression genes (DEGs) from GSE77803 which grouped by the expression of NUF2. Protein-protein interaction analysis (PPI, https://string-db.org/) for NUF2 was performed by STRING database (15). Molecular Complex Detection (MCODE) from Cytoscape was used to find clusters (highly interconnected regions) in a network. To further verify the enrichment analysis of the KEGG pathway, gene set enrichment analysis was performed using the GSEA program (v.4.0.3) (16). The data sets used include the H: hallmark gene set and C2.CP.KEGG (KEGG gene set) from the Broad Molecular Signatures Database (MSigDB) set. The number of random sample permutations is set to 1,000, and the significance threshold is P < 0.05.

### Basic Characteristic of NUF2 and Correlation Analysis Between NUF2 and Gene Markers

We first used the Genecards database (https://www.genecards.org/) and COMPARTMENTS (https://compartments.jensenlab.org/Search) database to know the genomic location and subcellular location of NUF2. The GEPIA database (http://gepia.cancer-pku.cn/index.html) was used to analyze the correlation between NUF2 and some special genes which maybe the genemarkers enriched in classic well-known pathway or cancer biomarkers, and also use it to understand the correlation between NUF2 expression and immune cell subset markers (17).

### NUF2 DNA Methylation Status and Mutation in NSCLC

We used the UALCAN database to evaluate the DNA methylation of NUF2 and the significance of NUF2 DNA methylation with clinical factors in NSCLC. And then, we used the c-Bio Portal database (http://cbioportal.org) to analyze NUF2 and the components of NDC80 complex alterations in TCGA NSCLC sample (18). The OncoPrint displays an overview alterations of genes above per sample in TCGA-NSCLC, while the mutations displays mutational site of every gene in detail. We also used the Comparison/Survival to explore the survival difference of NUF2 between gene altered group and unaltered group.

### Immune Correlation Analysis and Prognosis Analysis of NUF2 Based on Immune Infiltration

We next assessed whether the transcription levels of NUF2 in NSCLC were related to immune cell infiltration. TIMER database was used for correlation analysis (19). We use it to explore the association of NUF2 expression with immune cell (B cells, CD4 + T cells, NK cells, CD8 + T cells, Th1 cells, neutrophils, monocyte, macrophages, Treg, and dendritic cells, etc) infiltration and the subgroup markers of them. In order to describe the intensity of correlation between NUF2 expression level and tumor immune infiltrating levels in detail, we analyzed it under the guidance of absolute value: 0.00 to 0.19 “very weak,” 0.20–0.39 “weak,” 0.40 to 0.59 “moderate,” 0.60 to 0.79 “strong,” 0.80 to 1.0 “very strong.” We also further explore the prognosis analysis of NUF2 based on immune infiltration in NSCLC through Kaplan Meier plotter. TISIDB (http://cis.hku.hk/TISIDB/) was used to elucidate the interplay of NSCLC and immune cell subtype, immunoinhibitors, immunostimulators, and MHC molecules.

### RT-PCR

Take the adherent cell line with a good growth status and the growth area accounts for about 80% of the bottom of the bottle, using TRIzol reagent for total RNA extraction, and further transcribed into cDNA by reverse transcription. RT-qPCR was performed with SYBR qPCR mix (Takara Bio Inc) in a 7500 real-time PCR System (Thermo Fisher Scientific). GAPDH, whose primers were 5′-GTGGTCTCCTCTGACTTCAACA-3′(forward) and 5′-CTCTTCCTCTTGTGCTCTTGCT-3′(reverse), was regarded as an endogenous reference for normalization. The RT-PCR primers sequences of NUF2 were as follows: sense, 5′-TTTTTGCCTATCTGCCGGGT-3′(forward); and antisense, 5′-TGTGCGGCGTTTAACTGTTG-3′(reverse).

## Results

### Differential Expression of NUF2 in NSCLC

**Figure 1:**
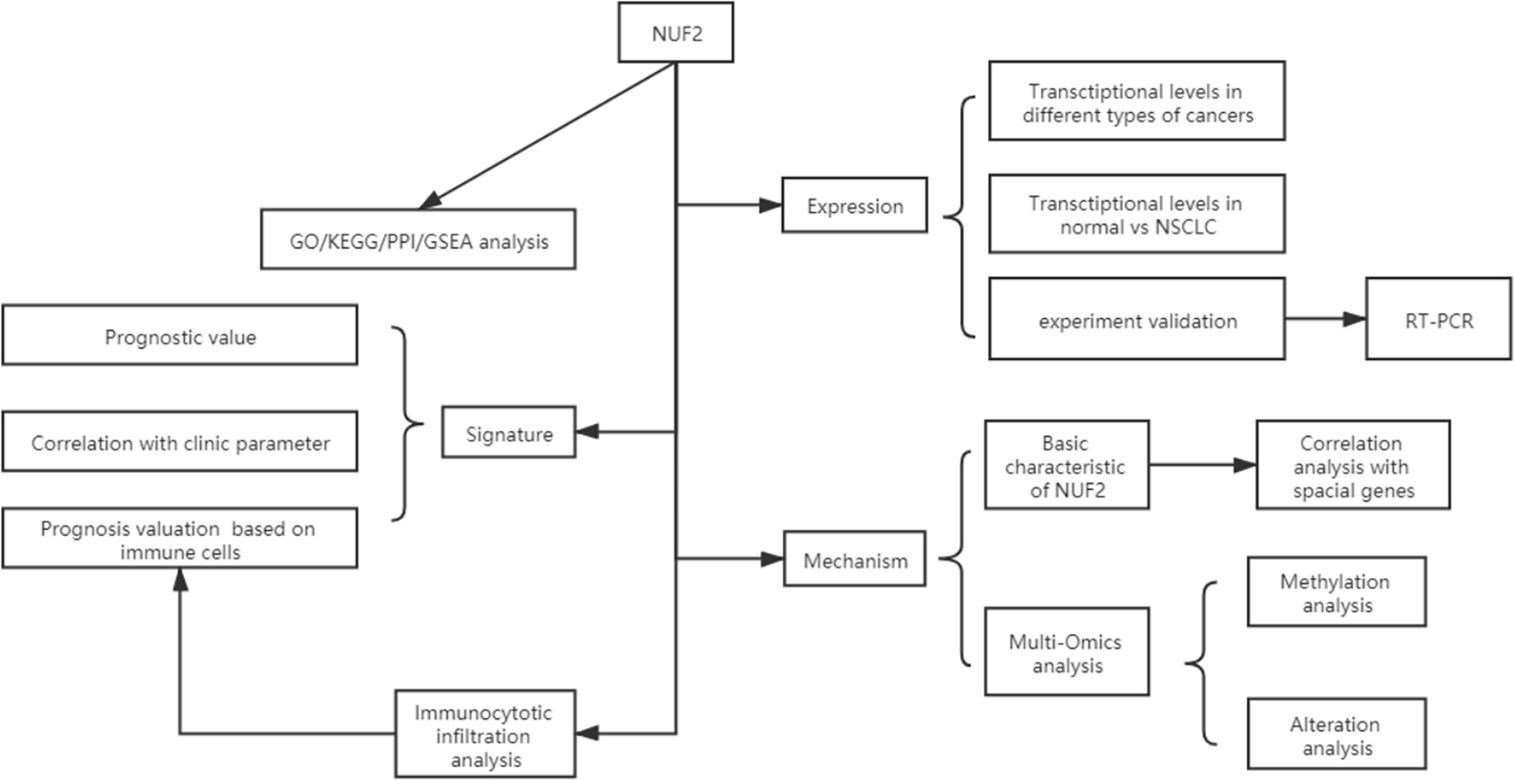
Flow diagram of data collection and method implementation in this work.

**Figure 2:**
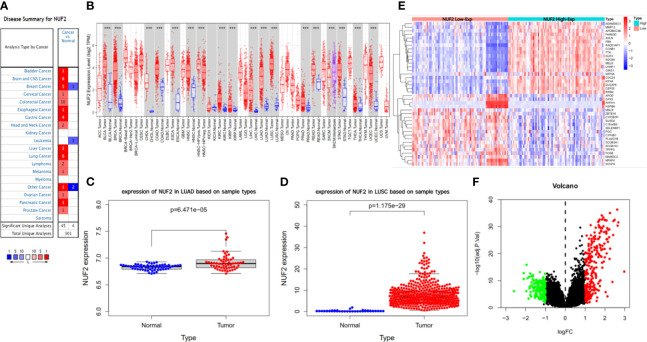
Difference analysis of transcription levels for NUF2 in different type of tumor tissues and normal tissues. **(A, B)** The expression level of NUF2 in different type of tumor tissues and normal tissues *via* Oncomine and TIMER. **(C, D)** The NUF2 expression level of tumor tissues and normal tissues in NSCLC from TCGA data set and GEO data set (GSE32863). **(E, F)** Heatmap and volcano plot of mRNA expression changes based on NUF2 gene expression in NSCLC samples from GSE77803. ***p < 0.001.

**Figure 3:**
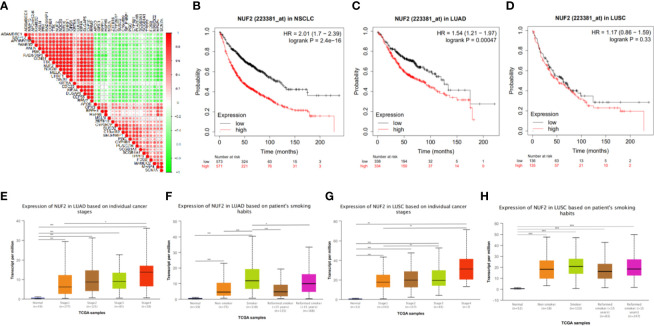
**(A)** The correlation analysis of TOP20 differentially upregulated and downregulated expressed genes. **(B–D)** Kaplan-Meier survival curve comparing the OS time for different NUF2 expression subgroups in NSCLC and the subtypes (LUAD and LUSC) of NSCLC. **(E–H)** The association of NUF2 expression with clincopathological factors. Expression of NUF2 in NSCLC with different stages and smoking habits based on TCGA sample *via* UALCAN. *p < 0.05, **p < 0.01, ***p < 0.001.

We developed a flow diagram to show our process (**Figure 1**). As shown in **Figures 2A, B**, data in Oncomine and TIMER revealed that mRNA expression of NUF2 were significantly higher in NSCLC than in normal tissue. NUF2 mRNA was also significantly over-expressed in GSE32863 (LUAD) and TCGA-FPKM (LUSC)(*P*<0.01) (**Figures 2C, D**). The volcano plot showed 262 genes (red dots) were significantly positive and 164 genes (green dots) were significantly negative correlated with NUF2, and the top 20 significant genes set positively and negatively correlated with NUF2 as shown in the heatmap (**Figures 2E, F**). Further, we assessed the association of the top 20 positive genes and negative gene in **Figure 3A**. All the results above demonstrated that NUF2 was a differential expression gene between NSCLC and normal lung tissue.

### The Association of NUF2 Expression With Prognosis and Clinical Features in NSCLC

We next explored the correlation between the transcription level of NUF2 with prognosis and clinical features. 1,144 patients with NSCLC divided into two groups according to the median transcription levels of NUF2, and we also analyzed the prognosis of LUAD (672 patients) and LUSC (271 patients) based on the median of NUF2 expression by Kaplan-Meier plotter. The results reveal that NUF2 high expression was significantly associated with a poorer OS in NSCLC (OS HR = 2.01, 95% CI = 1.7–2.39, logrank *P* = 2.4e−16) and LUAD (OS HR=1.54, 95% CI = 1.21–1.97, logrank *P* = 0.00047) (**Figures 3B, C**). However, there was no obviously significant connection between the expression of NUF2 and the OS of LUSC (**Figure 3D**). We further explored the association between NUF2 expression and clinical features. The NUF2 expression level of both LUAD and LUSC were higher in higher stages than lower stages (LUAD: stage 4 vs. stage 1; LUSC: stage 3 or 4 vs. stage 1) (**Figures 3E, G**). We also found the difference in subgroup of smoking habits (**Figures 3F, H**). These results showed that the NUF2 was significantly associated with clinicopathological factors in NSCLC.

### Functional Annotation and Protein-Protein Interaction

**Figure 4:**
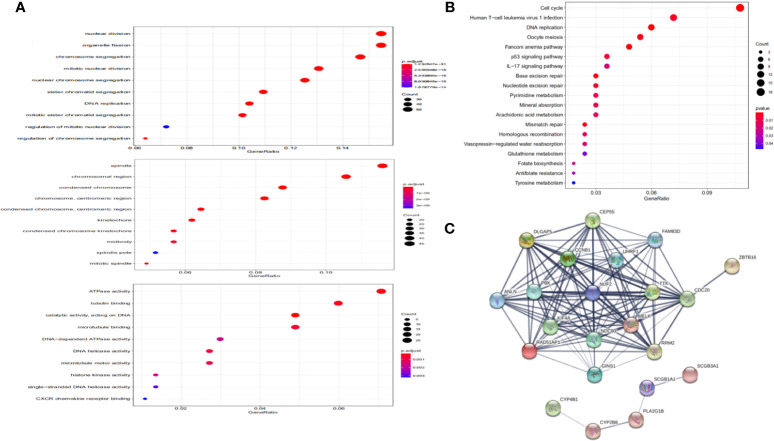
Functional annotation and protein-protein interaction. **(A)** Significantly enriched GO annotations of NUF2 in NSCLC. **(B)** Significantly enriched KEGG pathways of NUF2 in NSCLC. **(C)** The PPI network of NUF2 from STRING.

As shown in **Figure 4A**, DEGs were mainly enriched in organelle fission and nuclear division *via* Biological Process (BP) GO annotations, enriched in spindle *via* Cellular Component (CC) GO annotations and in ATPase activity *via* Molecular Function (MF) GO annotations respectively.

**Figure 5:**
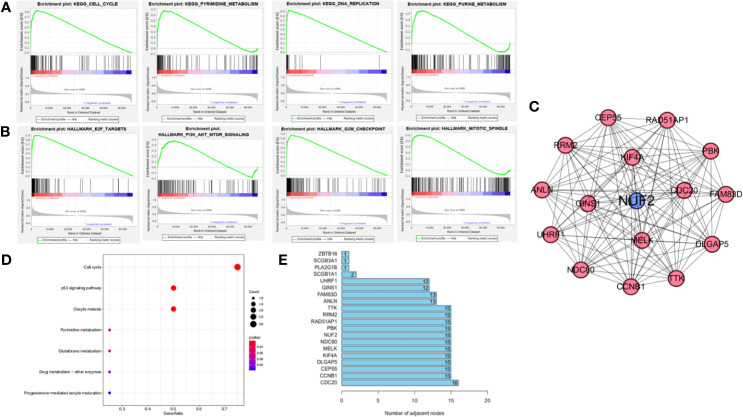
**(A, B)** Gene set enrichment analysis of NUF2 in KEGG and Hallmark collections. **(C)** The significant module *via* MCODE in the Cytoscape which contain the nodes with most edges. **(D)** The most significant pathway in the module *via* pathway enrichment analysis. **(E)** The number of edges about each node in the network.

KEGG pathway enrichment of NUF2 interactive genes showed that cell cycle was the most enriched pathways. Among them, the cell cycle had the smallest *P* value (*P* = 2.45e−10) and the largest number of involved consensus genes (count=18) (**Figure 4B**). As shown in **Figure 4C**, there were 22 nodes and 120 edges in the network of PPI *via* STRING. The vast majority of the nodes were up-regulated DEGs of NUF2 in the network. TTK, RRM2, RAD51AP1, PBK. NDC80, MELK, KIF4A, DLGAP5, CEP55, CCNB1, CDC20 have the most edges in the network (**Figure 5E**). In addition, we found a significant module *via* MCODE in the cytoscape and the most significant pathway in the module was also enriched in cell cycle (**Figures 5C, D**).

We further used GSEA enrichment analysis to demonstrate these results in MSigDB database. In curated gene sets (C2.CP.KEGG), the genes related with NUF2 have enriched in cell cycle, pyrimidine metabolism, purine metabolism and DNA replication; while in hallmark gene sets, the genes related with NUF2 have enriched in G2M checkpoint, E2F targets, mitotic spindle, PI3K-AKT-mTOR signaling (**Figures 5A, B**). Through functional enrichment analysis and PPI, we found that NUF2 may play an important part in cell cycle and the related genes of cell cycle.

### Basic Characteristic of NUF2 and the Correlation of NUF2 With Special Genes

**Figure 6:**
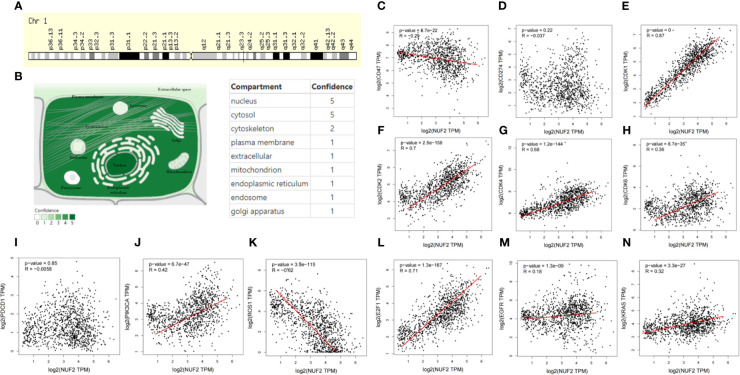
Basic characteristic of NUF2 and the correlation of NUF2 with special genes. **(A)** Genomic location of NUF2 gene *via* genecards. **(B)** Subcellular location and its confidence of NUF2 gene from COMPARTMENTS. **(C–N)** In the GEPIA database, scatterplots correlations of NUF2 with well-known oncogenes and genes related cell cycle.

NUF2 is a protein-coding RNA which located at q23.3 in Chromosome 1 (**Figure 6A**). In COMPARTMENTS, **Figure 6B** showed the subcellular locations of NUF2 and the highest confidence of subcellular locations were nucleus and cytosol. These results helped us to know the functional location of NUF2 in the cells. In order to better know the association of NUF2 with cell cycle and NSCLC, we explored the relationship between NUF2 and some special genes such as EGFR, KRAS, ROS1 and genes related with cell cycle by using GEPIA. We found that NUF2 was associated with KRAS, EGFR, ROS1, PIK3CA, and also related to CDK1/2/4/6, E2F1, which were enriched in cell cycle. NUF2 had a positive correlation with KRAS, EGFR, PIK3CA, CDK1/2/4/6, E2F1; while had a negative correlation with ROS1, CD47, but no correlation with CD274 and PDCD1 (**Figures 6C–N)**. By analyzing the relationship of NUF2 with cell cycle and tumor-related genes, it showed that NUF2 was involved in tumorigenesis and development.

### NUF2 DNA Methylation Status and Mutation in NSCLC

**Figure 7:**
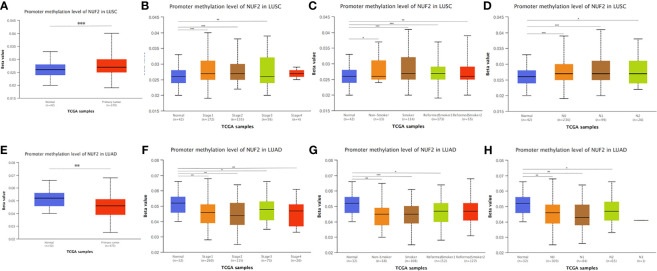
NUF2 DNA methylation status in NSCLC. **(A, E)** Promoter methylation level of NUF2 of tumor tissues and normal tissues in LUAD and LUSC using TCGA samples *via* UALCAN. **(B–D), (F–H)** The association of promoter methylation level of NUF2 with clincopathological factors: stages, smoking habits and N stage of AJCC TMN cancer stage. *p < 0.05, **p < 0.01, ***p < 0.001.

**Figure 8:**
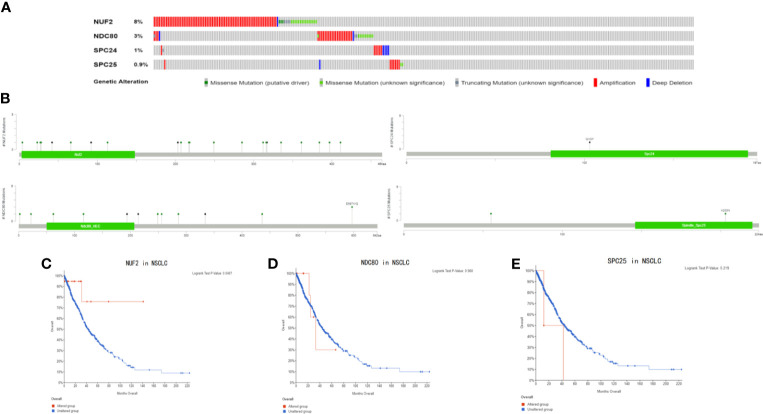
**(A)** The types and frequency of gene alterations of NUF2, NDC80, SPC24 and SPC25 gene. **(B)** Detailed mutation type of NUF2, NDC80, SPC24 and SPC25 gene in subtypes of NSCLC. **(C–E)** Kaplan-Meier survival curve comparing the OS time of NUF2, NDC80, SPC24 and SPC25 gene for different gene alteration subgroups in NSCLC.

**Figure 9:**
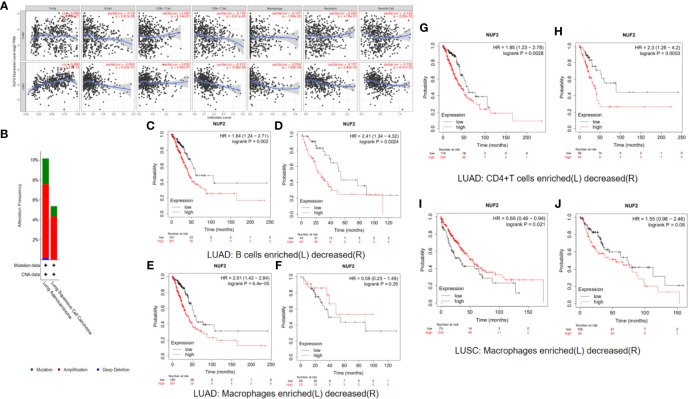
**(A)** Immune correlation analysis of NUF2 based on immune infiltration. NUF2 expression were negatively correlated with B cell, CD4 + T cell, Macrophage and dendritic cell immune infiltration levels of LUAD and also negatively correlated with CD4 + T cell, Macrophage, Neutrophil and dendritic cell immune infiltration levels of LUSC. **(B)** Gene alterations in detailed subtypes of NSCLC. **(C–J)** Kaplan-Meier survival curve comparing the OS time for different NUF2 expression in NSCLC based on immune cells infiltration.

In addition, we found that the promoter methylation level of NUF2 in LUSC was higher than that of normal tissues. In contrast, the promoter methylation level of NUF2 in LUAD was lower than in normal tissues (**Figures 7A, E**). Then, based on different clinical characteristics, we further discovered whether the promoter methylation level of NUF2 was correlated with clinical characteristics. The subgroup analysis results showed that the promotor methylation of NUF2 was possibly impact by stage, smoking status and N stage of AJCC TMN cancer stage (**Figures 7B–D, F–H**). We then used the cBioPortal to determine the types and frequency of NUF2 alterations based on whole exome sequencing data from NSCLC in TCGA (data including 42.3% LUSC and 57.7% LUAD). To highlight the role of NUF2 in NSCLC, we also compared NUF2 with NDC80, SPC24, SPC25, which were the component of NDC80 complex. The NDC80 complex was totally altered in 148 of 1,144 (12.9%) NSCLC patients, and most cases were NUF2-altered [92 of 1144 (8%)] (**Figure 8A**). The alteration of NUF2 accounts for 62% of the total alteration. Most of the gene alteration cases were gene amplification (**Figure 9B**). To go a step further, we explored the specific alteration of every genes, and we also found that the NUF2 has the most number of alteration sites in NSCLC than the others (**Figure 8B**). We next wanted to explore the correlation between alteration and the prognosis of NSCLC in cBioportal. 1144 patients were divided into two groups (altered group *vs*. unaltered group) according to the alteration of gene. As shown in **Figures 8C–E**, the altered group of NUF2 was significantly linked with a poor prognosis in somatic mutations, and we did not find obvious difference in the other genes of NDC80 complex. Although the NDC80 complex plays an important role in the tumorigenesis and development of the tumor cells, the above results may indicate that the mutation of the NUF2 molecule in the NDC80 complex may be related to the prognosis of NSCLC.

### Immune Correlation Analysis and Prognosis Analysis of NUF2 Based on Immune Infiltration

Previous studies have found that the immunoscores of lung squamous cell carcinoma and lung adenocarcinoma are significantly different, and the different tumor-infiltrating immune cell indicate different prognosis in LUSC and LUAD (20, 21). So we analyze immune correlation of NUF2 in LUSC and LUAD respectively. In LUSC, we have found that NUF2 was positively related with tumor purity weakly (cor = 0.388, *P* = 1.25e−18), negatively correlated with CD4+ T cells (partial.cor = −0.213, *P* = 2.96e−06) and macrophages weakly (partial.cor = −0.254, *P* = 1.88e−08), and negatively correlated with dendritic cells (partial.cor = −0.132, *P* = 4.01e−03) and neutrophils very weakly (partial.cor = −0.137, *P* = 2.83e−03). We also found that in LUAD, NUF2 was negatively correlated with B cells (partial.cor = −0.19, *P* = 2.67e−05), CD4+ T cells (partial.cor = −0.178, *P* = 8.61e−05), macrophages (partial.cor = −0.107, *P* = 1.89e−02), and dendritic cells very weakly (partial.cor = −0.133, *P* = 3.23e−03) (**Figure 9A**).

Markers of immune cells were considered to investigate further association of NUF2 expression with immune cells. No matter whether the correlation adjusted or not, NUF2 in LUSC was negatively correlated with markers of several immune cells, such as FCRL2, CD19, MS4A1 in B cells; FCGR3B, CEACAM3, SIGLEC5, FPR1, CSF3R, S100A12 in neutrophils; CD68, CD84, CD163, MS4A4A in macrophages; CD209, CD1C, CD141 in dendritic cells; FOXP3, CCR8 in Treg; C3AR1, CD86, CSF1R in monocyte (**Supplementary Table 1**). Meanwhile, NUF2 in LUAD was negatively correlated with MS4A1 in B cells, CD8A; CD8B in CD8+T cells; CSF3R, S100A12 in neutrophils, CD1C, CD141 in dendritic cells, KIR3DL3, NCR1 in NK cells; CSF1R in monocyte. We next used GEPIA to verify the results. In LUSC, correlation results between NUF2 and markers of macrophages, neutrophils, dendritic cells in GEPIA are similar to those in TIMER. In LUAD, correlation results between NUF2 and markers of B cells, dendritic cells CD8+T cells, NK cells in GEPIA are similar to those in TIMER (**Supplementary Table 2**).

We have demonstrated that the expression of NUF2 has some relationship with the immune cell infiltration in NSCLC, and the expression of NUF2 was also related to the prognosis of NSCLC. So we speculated whether the expression of NUF2 in NSCLC would have influence on the prognosis partly affected by immune cell infiltration. We analyzed the correlation between NUF2 expression and prognosis based on the enrichment of related immune cell in NSCLC. The results in LUAD revealed that higher expression of NUF2 in enriched B cells (HR=1.84, logrank P=0.002), enriched CD4+T cells (HR=1.85, logrank P=0.0028) and enriched macrophages (HR=2.01, logrank P=6.4e-05) had a poorer prognosis respectively (**Figures 9C–H**). The results in LUSC described that high expression of NUF2 in enriched macrophages have a better prognosis (HR=0.66, logrank P=0.021) (**Figures 9I, J**).

**Figure 10:**
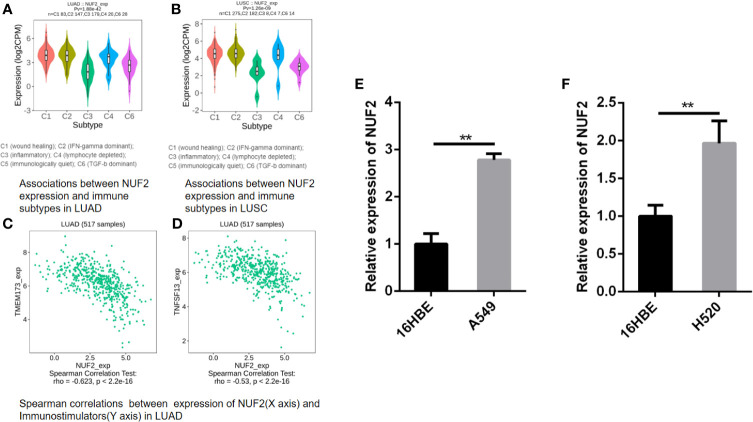
**(A, B)** Associations between NUF2 expression and immune subtypes in LUAD and LUSC. **(C, D)** Associations between NUF2 expression and immune subtypes in LUAD. **(E)** NUF2 expression was significantly higher in human lung adenocarcinoma cell line (A549) than in normal bronchial epithelial cells. **(F)** NUF2 expression was also significantly higher in human lung squamous cell line (H520) than in normal bronchial epithelial cells. **p < 0.01.

While multiple immune markers were found to be correlated with NUF2 expression in our study, we further want to explore NUF2 gene and other biomarkers in immune therapy through TISIDB. By dividing LUAD and LUSC into 6 groups respectively (eg. wound healing, IFN-gamma dominant, inflammatory, lymphocyte depleted, immunologically quiet, TGF-b dominant), we have found that the expression of NUF2 was the highest at wound healing (C1) and was the lowest at inflammatory (C3) in LUAD; while in LUSC, we have found that the expression of NUF2 was the highest at IFN-gamma dominant (C2) and also the lowest at inflammatory (C3) (**Figures 10A, B**). Furthermore, besides of TILs (Tumor Infiltrating Lymphocytes), MHC molecules, immunoinhibitors and immunostimulators were discovered the relationship with NUF2 expression in LUAD and LUSC through TISIDB (**Supplementary Figure 1**). After defining the thresholds as |r|>0.5 and p<0.01 to acquire effective immunomodulators, only TMEM173 and TNFSF13, as shown in immunostimulators of LUAD, these molecules was found to have a strong correlation with NUF2 expression (**Figures 10C, D**). This demonstrated that NUF2 may have the potential to impact the immune therapy induced by TMEM173 or TNFSF13 in LUAD. All these results indicated that NUF2 may have the potential to regulate immune infiltration and the response to immunotherapy, but these hypotheses need to be verified by further studies.

### Comparison of NUF2 mRNA Expression in Normal Human Bronchial Epithelial Cells and Lung Cancer Cell Lines

We further performed a qRT-PCR to demonstrate the expression level difference of NUF2 in GEO and TCGA. As shown in **Figures 10E, F**, both human lung adenocarcinoma cell line (A549) and human lung squamous cell carcinoma cell line (H520) expressed significantly higher levels of NUF2 than the normal human bronchial epithelial cells (16HBE).

## Discussion

With the appearance of modern genetics and molecular biology in the 1980s, researchers started to an gradually clear view of the genetic alterations that underlie uncontrolled proliferation in tumor cells (22–24). These disadvantage contain (but not limited to) the high expression of genes which drive or participate in cell cycle progression, such as cyclin D1 (CCND1), cyclin-dependent kinase 4 (CDK4) and cyclin-dependent kinase 6 (CDK6) (25–27). NUF2 is a composition of a molecular linker between the kinetochore attachment site and the tubulin subunits within the lattice of the attached plus ends (28). Although NUF2 was over-expressed in several cancers (5, 6), including lung adenocarcinoma (7), previous studies do not have analyzed NUF2 in lung adenocarcinoma from multi-omics aspects, and few study has analyzed this gene in squamous lung cancer.

In our study, we found that NUF2 was significantly higher both in LUAD and LUSC than in normal tissues. And we also verified the different expression levels of NUF2 mRNA between human normal bronchial epithelial cells and lung cancer cell lines *via* qRT-PCR, and demonstrated NUF2 mRNA expression were significantly higher in both human lung adenocarcinoma cell line and lung squamous cell line than in human normal bronchial epithelial cells. We have found that with the higher expression of NUF2, the patients had a poorer prognosis in LUAD but no difference in LUSC. This told us it might be a prognostic bio-marker in NSCLC and its subtype (LUAD), and the results which showed in clinical correlation analysis further indicated the impact of NUF2 in NSCLC. Through functional enrichment analysis, we knew that the NUF2 was significantly enriched in cell cycle, especially in DNA replication. Previous studies have found that the cyclin-dependent kinase (CDK)-RB-E2F axis was the key transcription mechanism that drives the cell cycle procession (29, 30). One or more important parts of this axis (cyclins, CDKs, CDK inhibitors and the RB family of proteins) are changed, which occurs in almost all cancers and leads to oncogenic E2F increased activity and uncontrolled proliferation (31). In our study, we found a significant association between NUF2 and these genes related to cell cycle procession or oncogenes in the progress of NSCLC, such as E2F1, CDK1/2/4/6, KRAS, EGFR, ROS1, PIK3CA. Therefore, NUF2 may be inferred to be a key gene involved in the occurrence, development, and prognosis of NSCLC.

The variation of the epigenome is thought to indicate the influence of genetic and environmental risk factors on multiple disease. DNA methylation is still the only epigenetic marker that can be stably detected in various samples (32). The change of normal DNA methylation patterns contain DNA hypomethylation, which occurs pathologically in normally unmethylated regions of the genome, and DNA hypermethylation, which usually occurs in the CpG islands of gene promoters (33). So far, previous studies have found a correlation between DNA methylation changes and many tumors (34, 35). In DNA methylation analysis, we found that NUF2 methylation level was lower in LUAD than in normal tissues. In contrast, we found that NUF2 methylation level was higher in LUSC than in normal tissues. The difference of NUF2 methylation levels in different types of NSCLC may suggest that the NUF2 gene has different epigenetic regulation between LUSC and LUAD. As there is no current research on NUF2 methylation, and our study could not perform experimental research for probing potential methylation mechanisms of NUF2 in NSCLC, further research was needed to explore the mechanism between NUF2 expression and NUF2 methylation in NSCLC, and whether the different levels of NUF2 methylation have different effects on the occurrence, development and prognosis between LUAD and LUSC, and to find the cause of the difference of NUF2 methylation between them.

Genome instability was the molecular genetic marker of tumorigenesis. And gene amplification, as the main form of genome instability, plays an important role in the occurrence and development of many human malignant tumors (36, 37). Few studies have explored the alteration of NUF2 in NSCLC. The NDC80 complex is a heterotetrameric protein complex that plays an important role in the process of cell mitosis and NUF2 is an component of it (26). In our study, we found that the alteration rate of NUF2 (8%) was higher than other components of NDC80 complex. We also found that NUF2 gene was mainly amplified in non-small cell lung cancer. These results suggested that in NDC80 complex, NUF2 gene amplification may play an important role in NSCLC. Due to the lack of research on NUF2 gene amplification, more research need to focus on the manifestations of NUF2 gene amplification in NSCLC, and the relationship between NUF2 gene amplification and NSCLC drug resistance or tumor cell escape growth inhibition. We also found that there was a significant difference between altered group and unaltered group of NUF2 in NSCLC, but no significance in NDC80 and SPC25. This may show that the NDC80 complex may affect the prognosis of NSCLC *via* NUF2 mutation, but further research is needed to confirm it.

A large number of studies have found a relationship between immune cells and tumor occurrence, development, and prognosis (38–40). Current research has found that some genes regulate tumor microenvironment through immune cells (41–43). So we explored the relationship between NUF2 and immune cell infiltration in NSCLC. Our results show that the association of NUF2 expression with the immune cell and its type marker implicate the negative impact of NUF2 in regulating tumor immunology in LUSC and LUAD. To go a step further, no matter in LUAD or LUSC, NUF2 gene was negatively correlated with macrophages and DCs. It reveals the potential regulating role of NUF2 in polarization of tumor-associated macrophages (TAM) and DCs. After that, the results of prognostic valuation based on immune cells showed a poorer survival rate of NUF2 high-expression in enriched macrophages both in LUAD and LUSC. These messages reminded us that NUF2 may affect the survival rate of NSCLC *via* TAM. In TISIDB, association of NUF2 with lymphocyte or immunemodulator also showed the negative relationship between NUF2 gene and immune infiltration. Further study on exploring the mechanism of NUF2 gene in immune infiltration should be added.

## Conclusions

To sum up, NUF2 transcription levels increased significantly in NSCLC. NUF2 was enriched in cell cycle *via* functional annotation and PPI analysis. In multi-omics analysis, we found that NDC80 complex may affect the prognosis of NSCLC *via* NUF2 mutation and NUF2 methylation level was lower in LUAD and higher in LUSC. Elevated NUF2 expression was negatively correlated with immune cells infiltration and prognosis of NSCLC, and NUF2 may affect the survival rate of NSCLC *via* TAM. Unfortunately, we could not perform experimental research for probing potential oncogenic mechanisms of NUF2 in NSCLC development. And, no our own follow-up data of NSCLC patients were available. In conclusion, our study revealed that the NUF2 gene may be a new prognostic marker of NSCLC, it may become an important molecular for the treatment of NSCLC. We also analyzed the role of NUF2 in NSCLC from multiple aspects. however, further studies need to be done to assess the diagnostic and therapeutic role of NUF2 in NSCLC.

## Data Availability Statement

The original contributions presented in the study are included in the article/**Supplementary Material**. Further inquiries can be directed to the corresponding authors.

## Author Contributions

Conception and design of the work: MC and WW. Acquisition, analysis and interpretation of data: MC, YL, YZ, and DL. Drafting and revising of the article: MC and WW, SL. Expression experiment: SL, MC. All authors contributed to the article and approved the submitted version.

## Conflict of Interest

The authors declare that the research was conducted in the absence of any commercial or financial relationships that could be construed as a potential conflict of interest.

## Supplementary Material

Supplementary Figure 1 Heatmaps show the spearman correlation between of NUF2 and lymphocyte/immunemodulator across human cancers.

## Abbreviations

NSCLC, non-small cell lung cancer; SCLC, small cell lung cancer; LUAD, lung adenocarcinoma; LUSC, lung squamous cell carcinoma; NDC80, the mitochondrial cycle 80 complex; KMplotter, Kaplan-Meier plotter; AJCC TNM, American Joint Committee on Cancer TNM stage; GO, gene ontology; KEGG, Kyoto Encyclopedia of Genes and Genomes; PPI, protein-protein interaction analysis; GSEA, gene-set enrichment analysis; MSigDB, the Broad Molecular Signatures Database set; c-BioPortal, cBio Cancer Genomics Portal; BP, biological process; CC, cellular component; MF, molecular function; MCODE, molecular complex detection; DCs, dendritic cells; CCND1, cyclin D1, CDK4, cyclin-dependent kinase 4; CDK6, cyclin-dependent kinase 6; CDK, cyclin-dependent kinase; TAM, tumor-associated macrophages.
