# Supplementary figures and images for: Integrative Multi-Omics Analysis of Identified NUF2 as a Candidate Oncogene Correlates With Poor Prognosis and Immune Infiltration in Non-Small Cell Lung Cancer
Source: Front Oncol. 2021 Jun 10;11:656509. doi: 10.3389/fonc.2021.656509 (PMC8222979; doi:10.3389/fonc.2021.656509)

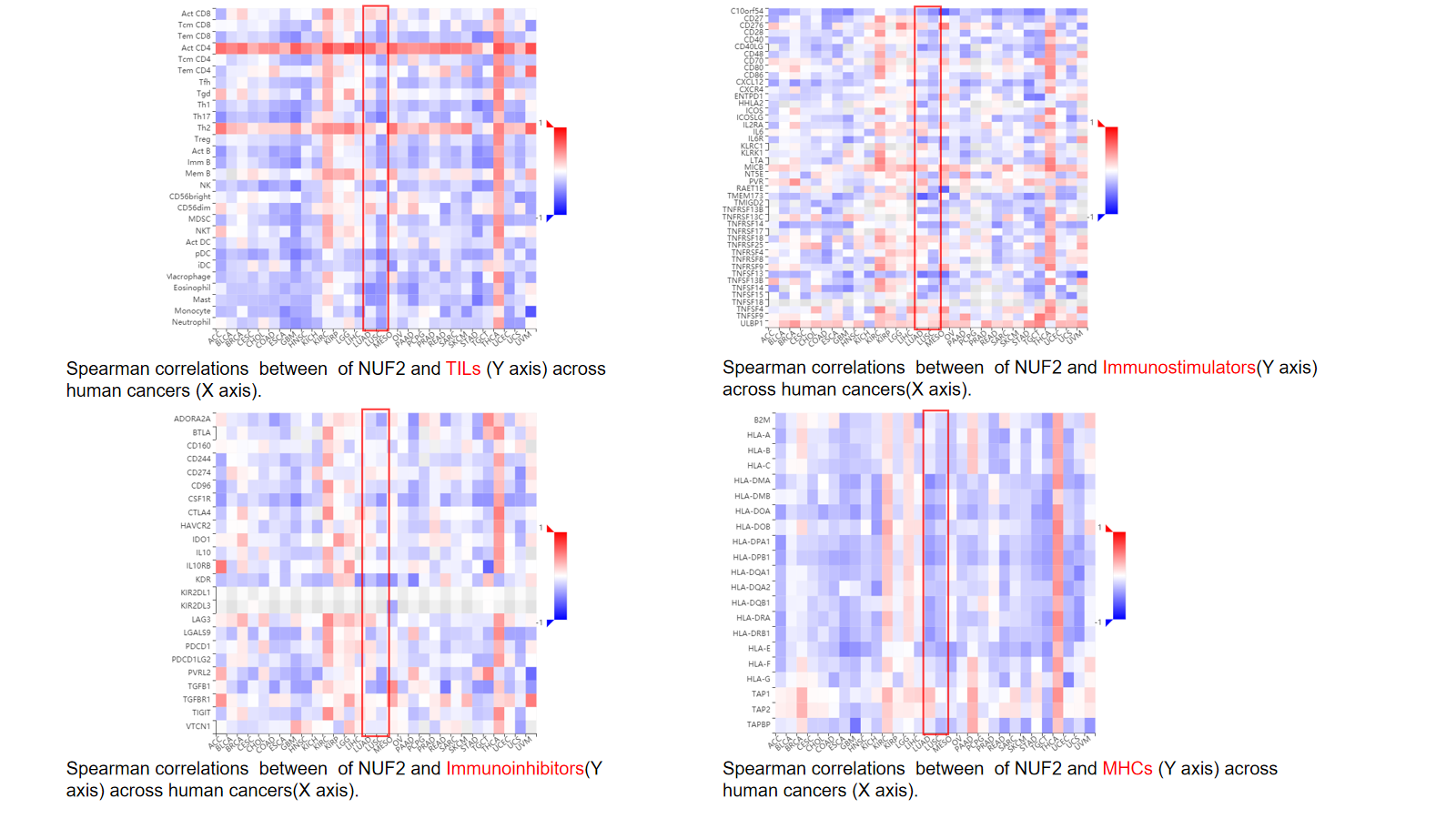

Supplement: Supplementary Figure 1 — Heatmaps show the spearman correlation between of NUF2 and lymphocyte/immunemodulator across human cancers. [file Image_1.tif]
