# Supplementary material for: Integrative Multi-Omics Analysis of Identified NUF2 as a Candidate Oncogene Correlates With Poor Prognosis and Immune Infiltration in Non-Small Cell Lung Cancer
Source: Front Oncol. 2021 Jun 10;11:656509. doi: 10.3389/fonc.2021.656509 (PMC8222979; doi:10.3389/fonc.2021.656509)
Supplement: Supplementary file 2 [file Table_1.docx]

**Supplementary Table 1.** Correlation results between NUF2 and markers of immune cells via TIMER

| Cell type | Gene  markers | LUAD | | | | | | | | | LUSC | | | | | | | | | |  |
| --- | --- | --- | --- | --- | --- | --- | --- | --- | --- | --- | --- | --- | --- | --- | --- | --- | --- | --- | --- | --- | --- |
|  |  | None | | | Purity | | | Age | | | | None | | | Purity | | | Age | | | |
|  |  | COR | P | COR | | P | COR | | P | COR | | | P | COR | | P | COR | | P |  |  |
| B cells | FCRL2 | -0.045 | 0.307 | -0.034 | | 0.450 | -0.038 | | 0.405 | -0.198 | | | 8.21E-06 | -0.178 | | 7.76E-05 | -0.198 | | 9.38E-06 |  |  |
|  | CD19 | -0.064 | 0.147 | -0.058 | | 0.200 | -0.0643 | | 0.158 | -0.215 | | | 1.25E-06 | -0.200 | | 1.02E-05 | -0.215 | | 1.58E-06 |  |  |
|  | MS4A1 | -0.163 | 0.0002 | -0.147 | | 0.001 | -0.159 | | 0.0005 | -0.164 | | | 0.0002 | -0.147 | | 0.001 | -0.168 | | 0.0002 |  |  |
| CD8+ T cells | CD8A | 0.137 | 0.002 | 0.140 | | 0.002 | 0.139 | | 0.002 | -0.005 | | | 0.910 | 0.006 | | 0.901 | -0.007 | | 0.879 |  |  |
|  | CD8B | 0.181 | 3.79E-05 | 0.176 | | 8.83E-05 | 0.181 | | 6.12E-05 | 0.068 | | | 0.131 | 0.080 | | 0.082 | 0.070 | | 0.1229 |  |  |
| Neutrophils | FCGR3B | 0.068 | 0.125 | 0.077 | | 0.086 | 0.068 | | 0.134 | -0.244 | | | 3.03E-08 | -0.234 | | 2.32E-07 | -0.242 | | 5.93E-08 |  |  |
|  | CEACAM3 | -0.082 | 0.064 | -0.080 | | 0.077 | -0.083 | | 0.067 | -0.322 | | | 1.59E-13 | -0.316 | | 1.64E-12 | -0.326 | | 1.18E-13 |  |  |
|  | SIGLEC5 | -0.086 | 0.050 | -0.090 | | 0.045 | -0.080 | | 0.078 | -0.289 | | | 4.23E-11 | -0.287 | | 1.63E-10 | -0.289 | | 6.80E-11 |  |  |
|  | FPR1 | -0.027 | 0.541 | -0.017 | | 0.699 | -0.019 | | 0.689 | -0.275 | | | 3.59E-10 | -0.266 | | 3.31E-09 | -0.278 | | 3.69E-10 |  |  |
|  | CSF3R | -0.144 | 0.001 | -0.146 | | 0.001 | -0.149 | | 0.001 | -0.327 | | | 5.55E-14 | -0.317 | | 1.21E-12 | -0.328 | | 7.79E-14 |  |  |
|  | S100A12 | 0.112 | 0.011 | 0.123 | | 0.006 | 0.116 | | 0.011 | -0.168 | | | 0.0002 | -0.185 | | 4.74E-05 | -0.167 | | 0.0002 |  |  |
| Macrophages | CD68 | -0.044 | 0.318 | -0.041 | | 0.354 | -0.038 | | 0.406 | -0.282 | | | 1.37E-10 | -0.281 | | 4.26E-10 | -0.274 | | 6.39E-10 |  |  |
|  | CD84 | -0.072 | 0.104 | -0.063 | | 0.159 | -0.070 | | 0.123 | -0.155 | | | 0.0005 | -0.147 | | 0.001 | -0.152 | | 0.0007 |  |  |
|  | CD163 | 0.002 | 0.970 | 0.003 | | 0.951 | 0.007 | | 0.872 | -0.257 | | | 5.04E-09 | -0.251 | | 2.85E-08 | -0.259 | | 6.06E-09 |  |  |
|  | MS4A4A | -0.074 | 0.093 | -0.069 | | 0.126 | -0.072 | | 0.110 | -0.232 | | | 1.54E-07 | -0.226 | | 5.64E-07 | -0.233 | | 1.81E-07 |  |  |
| Dendritic cells | CD209 | 0.032 | 0.465 | 0.031 | | 0.498 | 0.029 | | 0.526 | -0.242 | | | 3.95E-08 | -0.239 | | 1.24E-07 | -0.239 | | 8.25E-08 |  |  |
|  | CD1C | -0.52 | 4.31e-37 | -0.513 | | 2.01e-34 | -0.504 | | 1.51e-32 | -0.33 | | | 3.60e-14 | -0.167 | | 2.41e-04 | -0.32 | | 4.19e-13 |  |  |
|  | CD141 | -0.312 | 4.26e-13 | -0.31 | | 1.95e-12 | -0.294 | | 4.51e-11 | -0.197 | | | 9.34-06 | -0.191 | | 2.71e-05 | -0.187 | | 3.08e-05 |  |  |
| NK cells | KIR3DL3 | 0.187 | 1.97E-05 | 0.198 | | 9.24E-06 | 0.187 | | 3.43E-05 | 0.024 | | | 0.595 | 0.0286 | | 0.533 | 0.040 | | 0.376 |  |  |
|  | NCR1 | 0.131 | 0.003 | 0.137 | | 0.002 | 0.123 | | 0.007 | -0.042 | | | 0.346 | -0.040 | | 0.389 | -0.039 | | 0.386 |  |  |
| Th1 cells | TBX21 | -0.014 | 0.743 | -0.007 | | 0.872 | -0.012 | | 0.789 | -0.087 | | | 0.053 | -0.076 | | 0.096 | -0.084 | | 0.061 |  |  |
| Treg | FOXP3 | 0.015 | 0.731 | 0.017 | | 0.700 | -0.001 | | 0.987 | -0.183 | | | 3.79E-05 | -0.183 | | 5.61E-05 | -0.181 | | 5.49E-05 |  |  |
|  | CCR8 | 0.018 | 0.677 | 0.0256 | | 0.565 | 0.008 | | 0.861 | -0.163 | | | 0.0003 | -0.165 | | 0.0003 | -0.161 | | 0.0003 |  |  |
| Monocyte | C3AR1 | -0.063 | 0.154 | -0.055 | | 0.221 | -0.060 | | 0.186 | -0.227 | | | 2.92E-07 | -0.228 | | 6.92E-07 | -0.229 | | 2.73E-07 |  |  |
|  | CD86 | 0.007 | 0.871 | 0.0107 | | 0.813 | 0.008 | | 0.856 | -0.228 | | | 2.50E-07 | -0.229 | | 4.05E-07 | -0.228 | | 3.20E-07 |  |  |
|  | CSF1R | -0.160 | 0.0002 | -0.151 | | 0.0008 | -0.164 | | 0.0003 | -0.316 | | | 4.15E-13 | -0.320 | | 7.73E-13 | -0.317 | | 6.69E-13 |  |  |
