# Supplementary material for: Integrative Multi-Omics Analysis of Identified NUF2 as a Candidate Oncogene Correlates With Poor Prognosis and Immune Infiltration in Non-Small Cell Lung Cancer
Source: Front Oncol. 2021 Jun 10;11:656509. doi: 10.3389/fonc.2021.656509 (PMC8222979; doi:10.3389/fonc.2021.656509)
Supplement: Supplementary file 3 [file Table_2.docx]

| Cell type | Gene  markers | LUSC | | |
| --- | --- | --- | --- | --- |
|  |  | COR | | P |
| Macrophag-es | CD68 | −0.25 | 2.2e-08 | |
|  | CD84 | -0.2 | 6.6e-06 | |
|  | CD163 | -0.28 | 5.4e-10 | |
|  | MS4A4A | -0.25 | 4.3e-08 | |
| Neutrophils | FCGR3B | -0.21 | 2.5e-06 | |
|  | CEACAM3 | -0.3 | 2.1e-11 | |
|  | SIGLEC5 | -0.3 | 1.8e-11 | |
|  | FPR1 | -0.29 | 1e-11 | |
|  | CSF3R | -0.3 | 1.1e-11 | |
|  | S100A12 | -0.14 | 0.0018 | |
| Dendritic cells | CD209 | -0.24 | 1.2e−07 | |
|  | CD141 | -0.13 | 0.0029 | |
|  | CD1C | −0.33 | 7.2e−14 | |
| Cell type | Gene  markers | LUAD | | |
|  |  | COR | | P |
| B cells | FCRL2 | -0.07 | 0.13 | |
|  | CD19 | -0.077 | 0.09 | |
|  | MS4A1 | -0.18 | 8.5e-05 | |
| Macrophag-es | CD68 | -0.073 | 0.11 | |
|  | CD84 | -0.12 | 0.0096 | |
|  | CD163 | 0.024 | 0.59 | |
|  | MS4A4A | -0.099 | 0.03 | |
| Dendritic cells | CD209 | 0.0081 | 0.86 | |
|  | CD141 | -0.31 | 4.4e-12 | |
|  | CD1C | -0.51 | 1e-33 | |
| CD8+T cells | CD8A | 0.11 | 0.012 | |
|  | CD8B | 0.16 | 0.00028 | |
| NK cells | KIR3DL3 | 0.2 | 1.5e-05 | |
|  | NCR1 | 0.079 | 0.082 | |

**Supplementary Table** **2**. Correlation results between NUF2 and markers of immune cells via GEPIA
